# Assessment of ASHA for knowledge, diagnosis and treatment on malaria in Mandla district of Madhya Pradesh as part of the malaria elimination demonstration project

**DOI:** 10.1186/s12936-021-03610-8

**Published:** 2021-02-08

**Authors:** Harsh Rajvanshi, Kalyan B. Saha, Man Mohan Shukla, Sekh Nisar, Himanshu Jayswar, Ashok K. Mishra, Ravendra K. Sharma, Praveen K. Bharti, Nishant Saxena, Arvind Verma, Aparup Das, Harpreet Kaur, Suman L. Wattal, Altaf A. Lal

**Affiliations:** 1 Malaria Elimination Demonstration Project, Mandla, Madhya Pradesh India; 2 Indian Council of Medical Research-National Institute of Research in Tribal Health (ICMR-NIRTH), Jabalpur, Madhya Pradesh India; 3 Directorate of Health Services, Government of Madhya Pradesh, Bhopal, India; 4 Indian Council of Medical Research, Department of Health Research, Ministry of Health and Family Welfare, New Delhi, India; 5 National Vector Borne Disease Control Programme, Ministry of Health and Family Welfare, New Delhi, India; 6 Foundation for Disease Elimination and Control of India, Mumbai, Maharashtra India

**Keywords:** ASHA, Knowledge, Diagnosis, Treatment, Malaria, Training, Anti-malarials

## Abstract

**Background:**

The role of Accredited Social Health Activist (ASHA) in the health care delivery services at the periphery level is crucial for achieving disease prevention, control and elimination goals. The objective of the study was to assess the knowledge, attitude, practices, priorities and capability of ASHA related to malaria diagnosis and treatment as part of the Malaria Elimination Demonstration Project in 1233 villages of district Mandla, Madhya Pradesh.

**Methods:**

A cross sectional study was conducted using a fully structured, pre-tested interview schedule during June and July 2017 (before the field operations of MEDP were started). Two hundred twenty (17%) of the total ASHAs were selected for the interview from the 9 developmental blocks of Mandla district.

**Results:**

Knowledge, Attitude and Practices (KAP) study revealed that most ASHAs knew that mosquitoes are the main agent for spread of malaria (97.7%). They mostly used Rapid Diagnostic Test (RDT) for diagnosis (91.8%). The majority (87.3%) correctly identified negative RDT result while only 15% and 10.5%, respectively, identified *Plasmodium vivax* and *Plasmodium falciparum* positive cases correctly. Further analysis showed that 85% ASHAs used chloroquine, 44.5% used artemisinin-based combination therapy (ACT), and 55.5% used primaquine for treatment of malaria. It was also found that only 38.2% ASHA gave PQ for 14 days in cases of *P. vivax*. At the time of the interview, 19.1% ASHAs did not have any RDTs for diagnosis and 47.7% reported not having ACT for treatment of *P. falciparum* malaria.

**Conclusions:**

This study has revealed that ASHAs in the test district were not adequately trained or stocked for malaria parasite species identification and treatment, which are the major components of malaria elimination programme. This study has, therefore, revealed a need for training ASHAs on testing by RDT and proper treatment regimen for *P. vivax* and *P. falciparum*.

## Background

Malaria is a major public health problem in India, especially in rural/tribal areas of the country. India contributes ~ 80% of malaria cases in South East Asia Regional (SEAR) countries [1] and has the highest number of deaths outside the African continent, with approximately 1.2 billion at risk, including 183.5 million at high risk [1]. National Vector Borne Disease Control Programme (NVBDCP) has launched a national framework to eliminate malaria by 2030 [2]. The World Health Organization (WHO) and Roll Back Malaria (RBM) have also prepared materials and strategies for a malaria-free world by 2030 [3].

The National Health Mission (NHM) has focused specially on eight states that have limited infrastructure and low public health indicators. These eight Empowered Action Group (EAG) states are Bihar, Jharkhand, Madhya Pradesh, Chhattisgarh, Uttar Pradesh, Uttaranchal, Odisha and Rajasthan. One of the key components of the NHM is to provide one Accredited Social Health Activist (ASHA) who is a trained female community health activist for every 1000 population, which roughly translates to one ASHA for each village. The ASHA is selected from the village itself and accountable to it and she is trained to work as an interface between the community and the public health system.

ASHAs also act as health educators and promoters in their communities and they undergo series of modular training to acquire the necessary knowledge, skills and confidence for performing their spelled-out roles related to Maternal and Child Health (MCH), immunization, family planning, malaria and tuberculosis. Considering their reach and availability, they are also involved in diagnosis and treatment of malaria cases on a day-to-day basis. ASHA is supposed to screen suspected malaria cases, using Rapid Diagnostic Test (RDT) kits and blood slides and administer complete treatment as per the national drug policy [4, 5].

Malaria Elimination Demonstration Project (MEDP) is a first-of-its-kind public-private-partnership between the Indian Council of Medical Research (ICMR) through the National Institute of Research in Tribal Health (NIRTH), Government of Madhya Pradesh (GoMP), and the Foundation for Disease Elimination and Control of India (FDEC-India, established by Sun Pharmaceutical Industries Ltd. as a not-for-profit entity). The goal of MEDP is to demonstrate successful elimination of malaria from 1233 villages of Mandla district and use the lessons learnt for eliminating malaria from rest of Madhya Pradesh and the country.

The goal of MEDP is to demonstrate successful elimination of malaria from 1233 villages of Mandla district and use the lessons learnt for eliminating malaria from rest of Madhya Pradesh and the country. As a part of the Malaria Elimination Demonstration Project (MEDP), all ASHAs in project areas are engaged in malaria control strategies, like carrying out mass awareness, diagnosis and treatment of malaria cases.

A needs assessment on malaria for the Accredited Social Health Activists (ASHAs) of Mandla district was done to ensure that these village-based health care workers are informed and trained for malaria-specific community-based interventions, and to determine any training needs that would allow ASHAs to conduct their malaria elimination work in a reproducible and reliable manner.

## Methods

### Study setting

This study was carried out as part of the longitudinal Malaria Elimination Demonstration Project [6]. The district has an area of 8771 km², and is divided into nine development blocks, and 1233 villages. According to the 2011 census Mandla has a population of 1,053,522. Schedule Tribe (ST) is about 58% of total enumerated population and *Gonds* are primarily dominated tribe in the district.

### Study design

A cross sectional survey was conducted using a fully structured, pre-tested interview schedule. The ICMR guidelines 2017 for vulnerable groups were followed as Mandla district is predominantly tribal in nature [7]. The interview schedule was designed around: (1) Diagnosis and treatment; (2) Knowledge, Attitude, and Practices; (3) Vector control; (4) Incentives; and (5) Overall health literacy, and shared for review by the experts of the Malaria Elimination Advisory Group of MEDP Mandla [6]. Following their inputs, a pilot test run was conducted and the responses were analysed for consistency, validity, and measurability. To avoid bias, the subjects were chosen using random sampling, the tool had neutrally worded questions, and was structured in different sections to avoid overlapping of information. The schedule (Additional file 1) was administered by scientists and technical staff of ICMR-NIRTH Jabalpur and MEDP Mandla. The training done in May 2017 and involved a comprehensive review of the study-design followed by mock-interviews. The survey was done in the months of June and July 2017.

### Sampling technique and sample size

Out of the total 1300 ASHAs of Mandla district, 220 were selected for the interview. The sample size was estimated assuming: (1) 95% confidence; (2) 50% ASHAs had correct knowledge of malaria diagnosis and treatment; (3) 10% error; (4) design effect 2 and; (5) further inflated 15% non-response. Mandla consists of nine development blocks, namely Mandla, Narayanganj, Bijadandi, Ghughri, Mawai, Niwas, Mohgaon, Bicchia and Nainpur. The samples were drawn proportionally to number of ASHA working in a block, and within the block the ASHA were selected randomly using lottery method from the list of ASHAs provided by district health authorities.

### Ethical clearance

This study was part of the Malaria Elimination Demonstration Project, which has been cleared by the Institutional Ethical Clearance (IEC) Committee of ICMR—National Institute of Research in Tribal Health (NIRTH) on 16th March 2017 (reference no. 201701/10). An informed written consent was obtained from all participants.

### Data management and analysis

The data was entered in data entry software designed on CS-Pro 7.0 platform, and logical expressions and conditional statements were used to minimize the errors in data entry. The data analysis was done with Statistical Package for Social Sciences (SPSS) v25.0 (IBM SPSS Statistics, Armonk, NY: IBM Corp.) package. Univariate and bivariate analyses were performed. Means were compared using student ‘t’ test and p < 0.05  was considered for statistical significance.

Overall health literacy of ASHAs for malaria was assessed based on their awareness and perception on malaria’s sign and symptoms, mode of transmission, prevention, diagnosis and treatment. The health literacy was evaluated based on the following items: (i) how a person can get malaria, (ii) breeding sites of mosquitoes, (iii) who are at high risk of malaria, (iv) how one can avoid getting malaria, (v) common symptoms of malaria, (vi) how to diagnose malaria, (vii) minimum time limit for RDT kit to give correct result, (viii) correct identification of results from different RDTs pictures, (ix) correct identification of colour of ACT packs for different age groups, (x) identification of anti-malarial drugs, (xi) awareness about Insecticide-Treated Net (ITN)/Long-Lasting Insecticidal Net (LLIN), and (xii) number of days of administration of primaquine for treatment in case of *Plasmodium falciparum* and *Plasmodium vivax*.

Some of the questions had multiple correct answers, so overall these twelve questions had 35 correct responses. All correct responses were scored one (1) and for incorrect/ don’t know responses as zero (0). Scores for responses were added together to generate a literacy score, which varied from 4 to 29. These scores were converted to percentages ranging from minimum 11% to maximum of 83%. The percentage was categorized into three grades, namely low, medium and high, using cut-off values of < 40, 41–60 and > 60, respectively. The same technique was used for computing health literacy by Tobin et al. [8] and Muniyandi et al. [9].

## Results

### Socio‐demographic characteristics of respondents

 ASHAs were recruited from all the nine blocks of Mandla for this study and to maintain uniformity and obtain generalizability of results, equitable distribution of samples was obtained from all the blocks. The sampled ASHAs ranged between 13 in Mohgaon block to 33 in Bichhiya block of the district. The mean age of the ASHAs was 33.3 ± 5 years. About 98% of ASHAs were married and the mean number of children per ASHA was 2.3 ± 1.0. Majority of ASHAs belonged to Scheduled Tribes (ST) (66.8%) Other Backward Castes (OBC), Scheduled Castes (SC) and General categories at 25%, 6.4% and 1.8%, respectively. Regarding the education status, 47.7% ASHAs studied up-to middle school, 23.6% to high school, 12.3% to intermediate or higher, 12.7% to primary and 3.6% to grades lesser than Primary.

As per the guidelines by the National Health Mission, minimum educational qualification for recruitment of an ASHA is tenth grade or high school. This educational condition gets relaxed in situation where suitable person with prescribed qualification is not available in the village [10]. More than half of the ASHAs (59.2%) had worked for more than 10 years in this job, 20.6% for 5–9 years and 20.2% for less than 5 years.

### Training

**Fig. 1:**
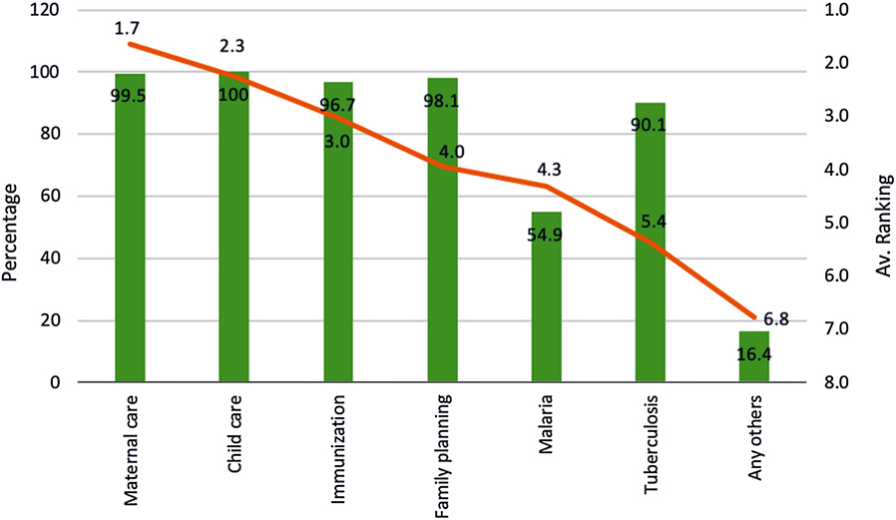
Percentage ASHAs received training and average ranking for activities. The green bars indicate the amount of training received in the respective area and the red line indicates the average priority given by ASHAs to the respective areas pertaining to various public health domains

ASHAs receive regular training for various components of their duties on a periodic basis. These trainings act as refreshers for the existing health programmes and inductive for the new ones. Almost all the ASHAs (99.5%) had received trainings on Maternal Health, but nearly half 54.9% on malaria. It was mentioned by the ASHAs that maternal care is their top priority service and malaria comes as number five out of seven priority areas (Fig. 1). ASHAs were trained by the state government on malaria tasks, which included preparing blood slide, rapid diagnostic test, and for prevention and treatment of malaria.

### Knowledge, Attitude and Practices (KAP) related to malaria

**Fig. 2:**
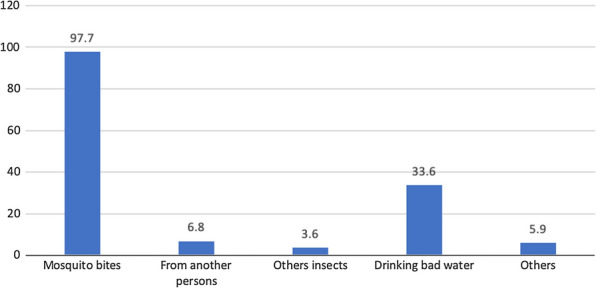
KAP related to Malaria: Spread of Malaria. The bar chart represents percentage of different reasons attributed to spread of Malaria by the 220 ASHAs interviewed

KAP assessment related to malaria was conducted for all the interviewed ASHAs. This assessment was crucial to identify the needs of ASHAs when it comes to diagnosis, treatment and prevention of malaria in the community. Most of the ASHAs knew about mosquitoes being the main agent for spread of malaria (97.7%). However, about 30% believed that malaria also spread by drinking bad water and various other misinformation for the spread of malaria (5.9%) (Fig. 2).

The majority of ASHAs (83.6%) knew that mosquitoes breed in stagnant water, however, about 12% reported fresh or running water as the site for breeding, while 3.2% informed garbage and 0.5% said dark and humid places. When asked about the method of diagnosing malaria, 91.8% ASHAs reported use of Rapid Diagnostic Test (RDT) kits, 85.9% also used blood slides, 8.2% mentioned diagnosed based on symptoms and 1.4% did not know how to diagnose it. Time limit to interpret results from the RDTs was told as 5 min by 41.4%, 15 min by 46.8%, 30 min by 8.6%, and 3.2% ASHAs did not know the answer to this question.

**Fig. 3:**
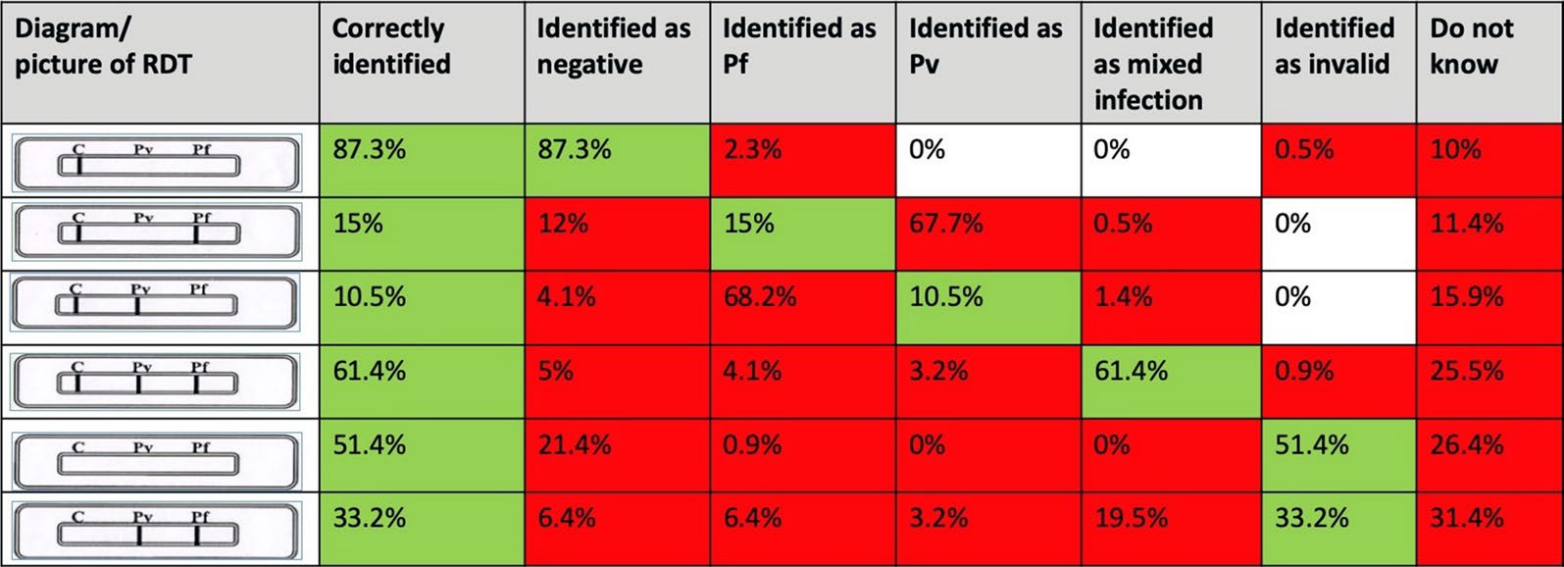
KAP related to Malaria: Interpretation of RDT result. The first column represents various scenarios of an RDT, the second column indicates percentage of ASHAs who correctly diagnosed the given scenario. Following these two columns, there are additional responses given by the participants. The ones highlighted in green and red are correct and incorrect/ undesirable, respectively - to the given scenario in column one

When ASHAs were asked to interpret various test results of the bivalent *P. falciparum/P. vivax* malaria RDT, except one, none could identify all the possible results correctly. Most (87.3%) correctly identified ‘negative’ result. *P. vivax* positive case was correctly recognized by only 15% ASHAs correctly and *P. falciparum* by 11%. About 60% could identify ‘mixed’ infections correctly and ‘invalid’ test results could be identified correctly by only 51.4% and 33.2% of the respondents (Fig. 3).

As per NVBDCP guidelines, chloroquine (CQ), primaquine (PQ) and artemisinin-based combination therapy (ACT) are the mainline recommended drugs for treatment of malaria. The study showed that 85% ASHAs used chloroquine, 44.5% ACT, 55.5% primaquine and 10% used other drugs for treatment of malaria. For the treatment of *P. falciparum*, PQ is given for one day only (2nd day) and for the treatment of *P. vivax*, PQ is given for 14 days. According to the responses received during the assessment, 52.7% and 41.8% did not dispense PQ in cases of *P. falciparum* and *P. vivax*, respectively. Whereas, 31.8% and 19.5% dispensed it for 13 days in cases of *P. falciparum* and *P. vivax*, respectively. 15% ASHAs gave PQ for 14 days in *P. falciparum*, which should have been given only once, and only 38.2% gave PQ for 14 days in cases of *P. vivax*.

Artemisinin-based combination is given for the treatment of uncomplicated *P. falciparum* malaria. It comes in different coloured packs for different age groups. The ASHAs were asked to identify these colored packs as recommended for specific age groups. The values highlighted in ‘yellow’ are the percentage of respondents who answered correctly. Nearly 25% ASHAs could recognize one correct ACT pack colour corresponding to the age group (Table 1). For age group 1–4 years and 15 + years, colour packs were commonly identified by the ASHAs. However, for other age groups very few could correctly identify.

**Table 1:**
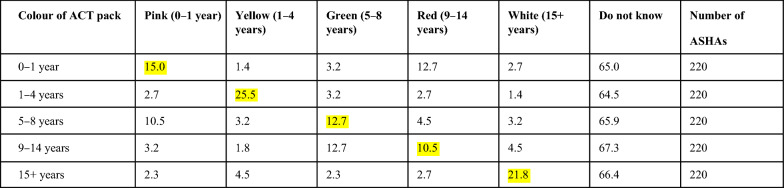
KAP related to treatment of malaria: Percentage of ASHAs (highlighted in yellow) who could correctly identify the color of the ACT pack with the corresponding age group

#### Fever cases attended to by ASHAs

Fever surveillance is the primary strategy to track, test and treat malaria cases in the community. On an average, maximum fever patients were covered by ASHA from Mohgaon block (31.3 in the last 3 months) and lowest of Mawai (8.9). and Mandla (5.1). It should be noted that Mawai is the block with highest number of cases of malaria. Preceding the survey, total average number of fever cases catered by each ASHA of entire Mandla district was observed to be 2.6, 6.3 and 13.4 in last week, last month and last 3 months, respectively (Table 2).

**Table 2:** Average number of fever cases seen by ASHAs of different blocks. The first, second and third columns depict the average fever cases seen in the last week, last month and last 3 months, respectively

Block	Population	Number of ASHAs	Number of ASHAs interviewed	Last week (fever cases)	Last month (fever cases)	Last 3 months (fever cases)
BICHHIA	175,385	274	33	2.7	7.1	13.2
BIJADANDI	79,358	132	25	2.3	6.4	17.4
GHUGHARI	108,235	103	21	6.1	8.1	16.6
MANDLA	231,313	164	27	2.6	3.5	5.1
MAWAI	113,630	154	36	1.2	4.8	8.9
MOHGAON	89,106	88	13	3.7	14.4	31.3
NAINPUR	175,876	157	28	2.3	5.5	10.7
NARAYANGANJ	92,782	130	21	2.5	6.1	16.6
NIWAS	77,441	98	16	1.6	5.0	11.2
Total	1,143,126	1300	220	2.6	6.3	13.4

#### Stock verification

A spot verification of stocks available with ASHAs was done. It was observed that 19.1% ASHAs did not have any RDTs with them. ACT packs for treatment of *P. falciparum* malaria were absent with almost half of the ASHAs (47.7%) and primaquine was also absent with almost half of ASHAs (46.4%) (Table 3).

**Table 3:** Stock verification of ASHAs. The first column indicates the various items in the stock followed by the quantity in percentage for each of those items. ‘N’ is total number of ASHAs

Stock item and status	Percentage of ASHAs			N
	Not available	Available	Not verified	
RDT	19.1	75.4	5.5	220
ACT	47.7	42.3	10.0	220
PQ	46.4	43.1	10.5	220
CQ	24.1	65.9	10.0	220
Others (anti-pyretic, ORS etc. )	54.1	32.7	13.2	220

#### Incentives

Out of the 220 ASHAs interviewed, 94% (207) said that they received monetary incentives during the last year preceding the survey. Out of which, 67% (138) mentioned that they have received it on-time. The receipt of highest incentive during the year preceding the survey and 3 months before the survey was reported highest in the block Narayanganj (19,529 INR/year and 4647 INR/3 months), while the incentive was least received at Nainpur block (5670 INR/year and 4095 INR/3 months). The average total incentive received by an ASHA of Mandla district was found to be 12,421 INR for last year and 3634 INR for last week.

#### Overall health literacy on malaria

Twelve questions with 35 correct responses were used to assess the level of literacy on malaria. Overall, the health literacy score varied from 4 to 29. Of the 220 ASHAs, 28.6% scored less than 40%, 53.2% scored between 41% and 60%, and only 18.2% scored more than 60%. The average health literacy scores according to respondent’s background characteristics are given in Table 4. The average health literacy score was significantly higher among older (40 or above years) ASHAs as compared to younger (less than 30 years) (18.8 vs. 15.4, p = 0.000). Similarly, middle and high school passed ASHAs had higher scores compared to illiterate/primary educated ASHAs. About 67% of interviewed ASHAs belonged to Scheduled Tribe (ST) communities, and by and large, they achieved considerably lower scores compared to Scheduled Castes (SC) and other castes.

**Table 4:** Mean and standard deviation of malaria literacy scores by background characteristics of ASHAs

Characteristics	Mean	SD	N (%)	P value
Age				
≤ 29	15.4	3.9	46 (20.9)	–
30–39	16.3	5.2	144 (65.4)	0.109
40+	18.8	4.0	30 (13.6)	0.000
Education				
≤Primary	13.4	4.6	36 (16.3)	–
Middle	15.6	5.1	105 (47.7)	0.024
High school	16.8	4.4	79 (35.9)	0.000
Caste				
ST	15.5	4.9	147 (66.8)	–
SC	17.8	4.3	14 (6.36)	0.092
Others (including general and other backward castes)	17.0	4.4	59 (26.8)	0.042
Primary occupation				
Home-maker	15.7	4.9	84 (38.1)	–
Own farming	16.9	4.8	51 (23.1)	0.167
Agriculture labour	15.2	4.6	51 (23.1)	0.557
Manual labour	16.4	4.4	26 (12.0)	0.516
Others	17.5	4.1	8 (3.7)	0.318
Rounds of training received				
≤ 3	13.2	4.0	21 (9.5)	–
4–6	16.7	5.3	72 (32.7)	0.006
7+	16.1	4.5	127 (57.8)	0.006
Received training on Malaria				
No	11.6	3.9	20 (9.0)	–
Yes	16.5	4.6	200 (91.0)	0.000
Total	16.0	4.8	220 (100)	

*SD* standard deviation,* N* total number of ASHAs

No specific trend was observed between malaria literacy scores and primary occupation of the ASHAs. Number of trainings had substantial positive impact on the health literacy scores compared to those received less training. ASHAs who had received more than 4 rounds of training had significantly higher malaria literacy scores. ASHAs received training on malaria had considerable higher scores (16.5 vs. 11.6, p = 0.000) compared to those who had not received training. The study shows that ASHA’s age, education, caste, and training had significant impact on their health literacy scores.

## Discussion

This study was performed to determine the needs-assessment of malaria diagnosis and treatment the ASHA workers of Mandla district. The findings of the study were grouped into various socio-demographic, training, incentives, knowledge, attitude, and practices (KAP) related to malaria, stock status, and overall health literacy indicators.

In this study, the mean age of the ASHAs complimented the mean ages of ASHAs in the other Indian states of Karnataka [11], Bihar (Samastipur) [12] and Odisha [13]. As per the guidelines of the National Health Mission, ASHA should be a literate woman with due preference in selection to those who are qualified up to 10th standard [5]. In the current study, 83.6% of ASHAs had completed at-least 8 years of schooling. In Karnataka, it was 90%, 82.6% in Bihar, and 85.75% in Odisha [12, 13]. The highest percentage observed in Karnataka may be due to the better literacy scores of the state [14].

Regarding the prioritization of different health programmes, the results of this study were supported by a study done by Fathima et al. in Karnataka [11], where more than 80% of the ASHAs reported MCH related work as their key activities and only 32% reported malaria as one of their important activities. However, a significantly higher readiness was observed in Odisha with 89% ASHAs trained in malaria diagnosis and treatment [13].

In this study, while majority of the ASHAs knew the method of diagnosis of malaria, the translation of this knowledge into practice was poor. Only 10% and 15% of the ASHAs could interpret the *P. falciparum* and *P. vivax* results on RDTs, respectively. These findings were contrary to a study done in Odisha, where the ability to interpret an RDT correctly was 86.8% [13], however, the unavailability of stock acted as an impediment in delivering services. In Wardha district of Maharashtra state, it was revealed that none of the ASHAs were taught about the diagnosis of malaria [15]. Another evaluation of three high endemic districts of Assam revealed that none of the ASHAs were involved in anti-malarial programme owing to lack of training and no supply of logistics [16]. In Mandla, the paradoxical combination of high-level awareness about malaria and lack of knowledge about the correct anti-malarial may be explained by a poor supply chain management system, where the ASHAs dispense the only available anti-malarial with them. The authors have demonstrated and discussed the makings of a sound supply chain management system used in the same district for malaria elimination [6].

ASHAs could not recognize correctly the age group-wise color packs of ACTs. However, with only 25% of ASHAs who could identify Yellow (1–4 years) ACT packs correctly. It was noticed that all ASHAs could not correctly identify the age-group of the colour-coded ACT combo-packs. Additionally, all ASHAs did not have adequate literacy levels to read the age-groups on the packs. It is recommended by the authors that along with the colour of packaging, the age-group may also be mentioned in the regional language on the packaging. It is also suggested that periodical assessments and refresher trainings of the ASHAs should be done similar to the methodology explained in a training study for malaria elimination in the same district [17].

## Conclusions

This study has revealed that ASHAs of Mandla district are not fully trained on malaria diagnosis and treatment. In the study district, most of ASHAs reported diagnosis of malaria through RDTs and blood slides, but only 10–15% could recognize *P. vivax* and *P. falciparum* RDTs correctly. ASHA’s awareness to ACT colour packs for different age group was also poor and PQ doses were not properly administered in case of *P. vivax* and *P. falciparu*m malaria cases. Lack of stock in abundance could also be a major barrier in diagnosing and administration of wrong type of anti-malarial to the patients. There is strong need for fresh and continuous training of ASHAs on RDTs and treatment regimen for *P. vivax* and *P. falciparum*, failing which, the goal of malaria elimination in India could be threatened.

## Supplementary Information

**Additional file 1.** ASHA needs-assessment tool for malaria elimination used in the study.

## Data Availability

We have reported all the findings in this manuscript. The hardcopy data is stored at MEDP Office in Mandla, Madhya Pradesh and Indian Council of Medical Research-National Institute of Research in Tribal Health (ICMR-NIRTH), Jabalpur, Madhya Pradesh. Softcopy data is available on the project server of MEDP hosted by Microsoft Azure. If anyone wants to review or use the data, they should contact: Dr. Altaf A. Lal. Project Director – Malaria Elimination Demonstration Project, Mandla. Foundation for Disease Elimination and Control of India, Mumbai, India 482003. E mail: altaf.lal@sunpharma.com.

## Abbreviations

ACT: Artemisinin-based Combination Therapy
ASHA: Accredited Social Health Activist
CQ: Chloroquine
EAG: Empowered Action Group
FDEC: Foundation for Disease Elimination and Control of India
GoMP: Government of Madhya Pradesh
ICMR: Indian Council of Medical Research
IEC: Institutional Ethical Committee
KAP: Knowledge, Attitude and Practices
MCH: Maternal and Child Health
MEDP: Malaria Elimination Demonstration Project
NHM: National Health Mission
NIRTH: National Institute of Research in Tribal Health
NVBDCP: National Vector Borne Disease Control Programme
OBC: Other Backward Caste
PQ: Primaquine
RBM: Roll Back Malaria partnership to end malaria
RDT: Rapid Diagnostic Test
SC: Scheduled Caste
SEAR: South East Asia Regional
SPSS: Statistical Package for Social Sciences
ST: Scheduled Tribe
WHO: World Health Organization
