# Supplementary material for: Assessment of ASHA for knowledge, diagnosis and treatment on malaria in Mandla district of Madhya Pradesh as part of the malaria elimination demonstration project
Source: Malar J. 2021 Feb 8;20:78. doi: 10.1186/s12936-021-03610-8 (PMC7871633; doi:10.1186/s12936-021-03610-8)
Supplement: Supplementary file 1 — Additional file 1. ASHA needs-assessment tool for malaria elimination used in the study. [file 12936_2021_3610_MOESM1_ESM.pdf]

CONFIDENTIAL
For Research and Malaria Elimination Demonstration Project Purpose Only

ASSESSMENT OF ASHA FOR KNOWLEDGE, DIAGNOSIS AND TREATMENT ON MALARIA IN MANDLA DISTRICT OF MADHYA PRADESH

ICMR-NATIONAL INSTITUTE FOR RESEARCH IN TRIBAL HEALTH, JABALPUR, GOVERNMENT OF MADHYA PRADESH, AND FOUNDATION FOR DISEASE ELIMINATION AND CONTROL OF INDIA (FDEC-India)

IDENTIFICATION	CODE
STATE (Name) : **MADHYA PRADESH**	
DISTRICT (Name) : **MANDLA**	☐☐
BLOCK (Name)________________	☐☐
HEALTH CENTRE (CHC/PHC) NAME : ________________	☐☐
SUB CENTER NAME/:________________	☐☐
VILLAGE (Name): ________________	☐☐
ASHA ID:	☐☐
Name of ASHA: ________________	
Date of interview:	☐☐ ☐☐ 1 7

Consent Form

We will be conducting a malaria elimination demonstration project in Mandla, which is a collaborative project between Govt. of MP, ICMR and FDEC. One of the essential components of the project is training of field workers involved in malaria programs. We are inviting you to participate in the study. The purpose of this study is to understand the ASHA's awareness regarding malaria, diagnosis and treatment at Mandla district of M.P. The results of this study will help us develop appropriate training modules.

There are no risks to participating in this study. Interview may take around 30-40 minutes. This will help in strengthening of ASHA's capability of handling malaria cases. There is no compensation for participation in this study. Your privacy is protected as a participant in this study.

If you have questions about this research you may contact Dr. Neeru Singh, Director, NIRTH (ICMR), Nagpur Road, Garha, Jabalpur, Madhya Pradesh on 0761-2370818/800.

Signature of the participant: ....

Signature of Investigator: ....

1. General Information to be collected from ASHA			
Questions		Response	Skip
101.	Is the sub-center located in this village?	Yes.....1<br>No.....2	
102.	Name of ASHA	Name :__________	
103.	Age in completed years of ASHA	Year [ ][ ]	
104.	Have you ever attended school?	Yes.....1<br>No.....2	If 2, go to 106
105.	If yes, what is the highest grade of education you completed?<br>***[Class I – XII =1 to 12 Grade; Bachelor's Degree = 15 Grade; Master's Degree=17 Grade; Engineering=16 Grade; MBBS =14 Grade; Polytechnic=13 Grade; ITI=11 Grade; Ph.D.= 20 Grade]**	Grade* [ ][ ]	
106.	What is your current marital status?	Not Married.....1<br>Ever Married.....2	If 1, go to 109
107.	CHECK: If ASHA is currently married i.e. answer of 106 is 2 and is currently married, what is the level of education of (ASHA) husband?<br>***[Class I – XII =1 to 12 Grade; Bachelor's Degree = 15 Grade; Master's Degree=17 Grade; Engineering=16 Grade; MBBS =14 Grade; Polytechnic=13 Grade; ITI=11 Grade; Ph.D.= 20 Grade]**	Grade* [ ][ ]	
108.	How many children do you have?	Number [ ][ ]	
109.	What is your caste/ tribe?<br>Caste/ tribe (Name).....	SC.....1<br>ST.....2<br>OBC.....3<br>GEN.....4	
110.	Other than ASHA, what else you do for earning/other source of earning?	Nothing else (Only housewife) .....1<br>Work as agricultural labour .....2<br>Other manual labour .....3<br>Work in the own farm .....4<br>Small trade (Shop, etc.) .....5<br>Forest produce.....6<br>Others (Specify).....8	

111.	CHECK: If ASHA is currently married i.e. answer of 106 is 2 and is currently married, What is the main work/occupation of your husband?	Work as agricultural labour ....1<br>Other manual labour......................................2<br>Work in the own farm ....3<br>Small trade (Shop, etc.) ....4<br>Forest produce ....5<br>Others (Specify) 8	**If 2 in Q 106, Ask this**
112.	Since how long are you working as ASHA?	Year [ ][ ]<br>Months [ ][ ]	
113.	Have you received any training as an ASHA?	Yes..1<br>No...2	**If no, go to 119**
114.	If yes, how many rounds with number of days?	Rounds: 1 | 2 | 3 | 4 | 5 | 6 | 7<br>No. of Days:	
115.	Have you been trained in any of the following?<br>**[Read all options]**<br>**(If training received only from Govt. agency write 1, only from Pvt. agency /NGO write 2, if from both Govt. & Pvt./NGO write 3).**	(Yes=1/ No=2 | Agency)<br>Maternal care?<br>Child care and feeding?<br>Family planning?<br>Immunization?<br>Malaria?<br>Tuberculosis (TB)<br>Any Other ________	
116.	If training received from any Pvt. agency/NGO, kindly specify the name(s)	1. ____________<br>2. ____________<br>3. ____________<br>4. Don't Know____________	
117.	If training received on malaria, what was the duration?	Rounds: 1 | 2 | 3 | 4 | 5 | 6 | 7<br>No. of Days:	
118.	What was the place of training?	1. ____________<br>2. ____________<br>3. ____________<br>4. Don't Know____________	
119.	Do the people of this village(s) come to you when they fall ill?	Yes..1<br>No...2	**If no, go to 121**
120.	If yes, how many patients have you seen?	Last week (numbers) [ ][ ]<br>Last Months (numbers) [ ][ ]	

121.	Do you motivate pregnant women for institutional delivery?	Yes..1<br>No...2	
122.	Do you take pregnant women for institutional delivery?	Yes..1<br>No...2	**If no, go to 124**
123.	If yes, how many pregnant women you have taken for institutional delivery?	Last week (numbers) ☐☐<br>Last Months (numbers) ☐☐	
124.	How do you motivate people to visit you for health seeking?	Door to door campaign...........................1<br>Inter-person communication ....2<br>Participate through Govt. Programs........3<br>Others (specify) 4	
125.	Do you have any written information on malaria which can be shown to the people for awareness and motivation?	Yes..1<br>No...2	
126.	In case of any confusion related to malaria, whom do you contact?	....<br>..<br>..<br>..	
127.	According to you, what are the important works that you presently do as ASHA?<br>**[Don't read the options, rank according to her response, starting with 1 for first response].**	**Rank**<br>Maternal care ? ☐<br>Child care and feeding ? ☐<br>Family planning? ☐<br>Immunization? ☐<br>Malaria? ☐<br>Tuberculosis (TB) ? ☐<br>Any Other _______ ☐	
128.	Have you ever received any payment/ incentives for your work as ASHA?	Yes ....1<br>No 2	**If no, go to 201**
129.	Do you receive payment/incentives in time?	Yes ....1<br>No 2	
130.	How much payment/incentives have you received for working as an ASHA?	Last 1 year Rs. ☐☐☐☐☐<br>Last 3 months Rs. ☐☐☐☐☐<br>Don't know 99999	

2. Now I will ask some questions related to your Knowledge on Malaria			
201.	Do you know how a person can get malaria?<br><br>**[Do not read or prompt. Circle all that are mentioned. Keep asking, "Anything else?"]**	Mosquito bites ....1<br>Microorganism ....2<br>(plasmodium / vivax / falciparum)<br>From another person..................................3<br>Other insects ....4<br>Drinking bad water.......................................5<br>Other (specify) ....8<br>Don't know 9	
202.	Breeding of malaria causing mosquito takes place in ?	Stagnant water..1<br>Fresh or running water.............................2<br>Garbage...3<br>Dark and humid place...............................4	
203.	Which malaria species is commonly found in your area?	*Plasmodium vivax* ....1<br>*Plasmodium falciparum*..........................2	
204.	Which is the high risk group for malaria disease?	Infants and pregnant women...................1<br>Old age group..2<br>children of 2-10 years...............................3<br>All of the above...4	
205.	Do you know how one can avoid getting malaria?<br>[Do not read or prompt. Circle all that are mentioned. Keep asking, "Anything else?"]	Use mosquito nets ....01<br>Use ITN/LLIN ....02<br>Clear stagnant water................................03<br>Use insecticide spray ....04<br>Use repellents (coils, etc).........................05<br>Use smoke...06<br>Use skin lotions or ointments (oils, etc) to keep away mosquitoes.............................07<br>Wear clothes to cover the body..............08<br>Take medicines for malaria.....................09<br>Keep surroundings clean........................10<br>Other (specify)..88<br>Don't know 99	

206.	Do you know what the common symptoms of malaria are?<br><br>**[Do not read or prompt. Circle all that are mentioned. Keep asking, "Anything else?"]**	High fever ....1<br>Ordinary fever ....2<br>Chills ....3<br>Headache ....4<br>Bodyache ....5<br>Nausea/vomiting....................6<br>Other (specify)....................8<br>Don't know 9	
**3. Now I will ask questions related to your knowledge on diagnosis of malaria**			
301.	Do you diagnose and provide any treatment to patients with fever?	Yes....................1<br>No....................2	**If no, go to 304**
302.	If no, why	....................<br>....................<br>....................<br>....................	
303.	If no, where do you refer these cases?	MPW....................1<br>Sub-Centre....................2<br>PHC/CHC....................3<br>Volunteer....................4<br>Pvt. Health post....................5	
304.	Do you receive fever patents referred by others?	Yes....................1<br>No....................2	**If no, go to 306**
305.	If yes, who refer fever cases to you?	Villages/PRI member....................1<br>Volunteers....................2<br>Local Traditional Healers....................3<br>Others 8	
306.	How do you diagnose malaria presently? **[Multiple Responses Possible]**	RDT....................1<br>Blood slide....................2<br>Symptomatic....................3<br>Don't diagnose....................4	**Do no ask if Q 301 is No.**
307.	What are the items required for slide preparation for diagnosing of malaria?<br>(tell one by one)	....................<br>....................<br>....................<br>....................<br>....................<br>....................<br>....................<br>....................	

308.	How the blood slides are prepared?<br>(tell step wise)	……………………………………………………<br>……………………………………………………<br>……………………………………………………<br>……………………………………………………<br>……………………………………………………<br>……………………………………………………<br>……………………………………………………<br>……………………………………………………<br>……………………………………………………	
309.	How can you diagnose malaria by RDT kit? (describe the method)	……………………………………………………<br>……………………………………………………<br>……………………………………………………<br>……………………………………………………<br>……………………………………………………<br>……………………………………………………<br>……………………………………………………<br>……………………………………………………<br>……………………………………………………<br>……………………………………………………	
310.	What is the minimum time limit for RDT kit to give correct result?	5 minute……………………………………1<br>15 minutes…………………………………2<br>30 minutes…………………………………3<br>45 minutes…………………………………4<br>60 minutes…………………………………5	
311.	What do you understand by the following diagrams?		

	a) 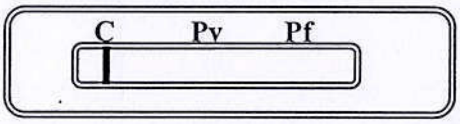		
	b) 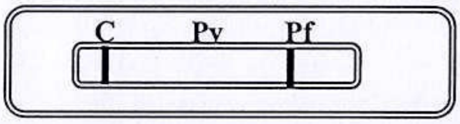		
	c) 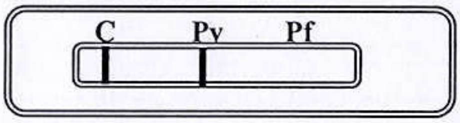		
	d) 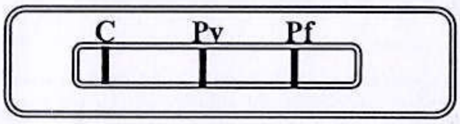		
	e) 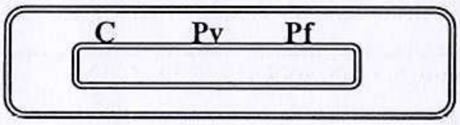		
	f) 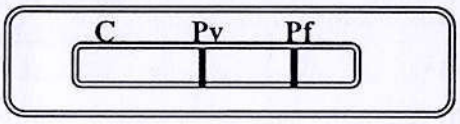		
312.	If you get invalid result (No "C" line) in RDT kit test then what you do next?	....<br>..<br>..<br>..<br>..<br>..<br>..<br>..<br>..	
313.	How do you transport slide/ RDT to the laboratory, once prepared?	....<br>..<br>..<br>..	**Do not ask if Q 301 is No.**
314.	In how many days, after preparation, you transport the slides/ RDT?	☐☐ Days	**Do not ask if Q 301 is No.**
315.	What format do you use to report malaria positive cases to higher centre?	....<br>..<br>..<br>..	**Do not ask if Q 301 is No.**

316.	How do you send the format to higher centre?	......................................<br>......................................<br>......................................<br>......................................	Do not ask if Q 301 is No.
**4. Now I will ask questions related to your monthly target for fever cases, source & quantity of procurement of antimalarial kits/drugs and knowledge on treatment for malaria (Ask only to those where Q 301 is Yes)**			
401.	What is the per month target fever cases you have to cover?	Cases [ ][ ][ ]	
402.	If the fever cases exceed the target in a month, how you cope up with the situation?	......................................<br>......................................<br>......................................<br>......................................	
403.	What are the antimalarial drugs with which you treat malaria presently? **[Multiple Responses Possible]**	Chloroquine........................1<br>ACT........................2<br>Priamaquine........................3<br>Others (Specify)........................4<br>Refer........................5<br>Don't treat/refer........................6	
404.	Who provide you medicines for service/illness?	Dot not get medicine........................1<br>ANM........................2<br>MPW(M)/MPHW........................3<br>Health sub-centre........................4<br>Others source (specify) 8	
405.	How many RDTs and Antimalarial drugs do you receive per month?	Total Quantity<br>RDT [ ]<br>ACT [ ]<br>Primaquine [ ]<br>Chloroquine [ ]<br>Others ________ [ ]	
406.	What is the treatment for Plasmodium vivax (Pv) and Plasmodium falciparum (Pf)? (explain in detail)	For Pv cases:........................<br>......................................<br>......................................<br>......................................<br>For Pf cases:........................<br>......................................<br>......................................<br>......................................	

407.	Match the following for treatment of *P. falciparum*	(1)....0 – 1 Green ☐<br>(2)....1 – 4 White ☐<br>(3)....5 – 8 Red ☐<br>(4)....9 – 14 Pink ☐<br>(5)....15 and above Yellow ☐	
408.	For treatment of Pv cases, do you give Primaquine?	Yes..1<br>No...2	**If no, go to 410**
409.	If yes, for how many days?	Days ☐☐	
410.	For treatment of Pf cases, do you give Primaquine?	Yes..1<br>No...2	**If no, go to 501**
411.	If yes, for how many days?	Days ☐☐	
**5. Now I will ask questions related to actually malaria cases diagnosed, treated and incentives received by you**			
501.	Do you record cases of fever that come to you?	Yes..1<br>No...2	**If no, go to 505**
502.	If yes, how many fever cases have you seen?	Last week (numbers) ☐☐<br>Last Months (numbers) ☐☐<br>Last 3 Months (numbers) ☐☐	
503.	How many malaria positive cases you diagnosed	**P. v** / **P. f**<br>Last week (numbers) ☐☐<br>Last Months (numbers) ☐☐<br>Last 3 Months (numbers) ☐☐	
504.	How many malaria positive cases you treated	**P. v** / **P. f**<br>Last week (numbers) ☐☐<br>Last Months (numbers) ☐☐<br>Last 3 Months (numbers) ☐☐	
505.	What will you do for malaria negative cases ? (explain)	....<br>..<br>..	

506.	Do you receive any payment/incentives for diagnosis and treatment of malaria cases?	Yes……………………………………1<br>No……………………………………2	**If no, go to 601**
507.	How much payment/incentives have you received for diagnosis and treatment of malaria cases?	Last 3 months Rs. [ ][ ][ ][ ][ ]<br>Last 1 year Rs. [ ][ ][ ][ ][ ]<br>Don't know 99999	
**6. Now I will ask questions on your knowledge on bednets**			
601.	Mosquito nets are distributed in your village (s)?	Yes……………………………………1<br>No……………………………………2	**If no, go to 603**
602.	If yes, when the nets are distributed?	Recently Days [ ][ ]<br>Months back (numbers) [ ][ ]<br>Year back (numbers) [ ][ ]<br>Don't know 99 [ ][ ]	
603.	Do you know what is ITN/ LLIN?	Yes……………………………………1<br>No……………………………………2	
**7. Stock verification on the date of survey**			
701.	**Please check and record all available malaria diagnostics and anti-malarial medicine available with ASHA**	Total Quantity<br>RDT [ ]<br>ACT [ ]<br>Primaquine [ ]<br>Chloroquine [ ]<br>Others _______ [ ]	
702.	Do you run out of anti-malarial drugs and RDTs?	Yes……………………………………1<br>No……………………………………2	**If yes, go to 703**
703.	If yes, for how many days/ months you operate without anti-malarial drugs/ RDTs	Anti-malarial drugs : ……………… Days<br>RDTs : ……………… Days	
704.	When you run out of anti-malarial drugs/ RDTs, who do you inform?	……………………………………<br>……………………………………<br>……………………………………	
705.	When you inform? **(Refers to duration of information)**	Same day……………………………………1<br>Next day……………………………………2<br>Within a week……………………………………3<br>More than a week……………………………………4	
